# A cohort study of short-term functional outcomes following injury: the role of pre-injury socio-demographic and health characteristics, injury and injury-related healthcare

**DOI:** 10.1186/1477-7525-9-68

**Published:** 2011-08-18

**Authors:** John Langley, Sarah Derrett, Gabrielle Davie, Shanthi Ameratunga, Emma Wyeth

**Affiliations:** 1 Injury Prevention Research Unit, Department of Preventive and Social Medicine, University of Otago, 55 Hanover St, Dunedin 9054, New Zealand; 2 Section of Epidemiology & Biostatistics, School of Population Health, Faculty of Medical and Health Sciences, University of Auckland, 261 Morrin Road, Glen Innes, Auckland 1072, New Zealand; 3 Ngāi Tahu Māori Health Research Unit, Department of Preventive and Social Medicine, University of Otago, 18 Frederick St, Dunedin 9054, New Zealand

**Keywords:** injury, short-term function, EQ-5D, outcomes, health status, quality of life

## Abstract

**Background:**

Injury outcome studies have tended to collect limited pre-injury characteristics, focus on a narrow range of injury types, predictors and outcomes, and be restricted to high threat to life injuries. We sought to identify the role of pre-injury socio-demographic and health characteristics, injury and injury-related healthcare in determining short-term functional outcomes for a wide range of injuries.

**Methods:**

Study participants (aged 18-64 years inclusive) were those in the Prospective Outcomes of Injury Study, a cohort of 2856 persons who were injured and registered with New Zealand's national no-fault injury insurance agency. All information used in this paper was obtained directly from the participants, primarily by telephone interviews, approximately three months after their injury. The functional outcomes of interest were the five dimensions of the EQ-5D plus a cognitive dimension. We initially examined bivariate relationships between our independent measures and the dependent measures. Our multivariate analyses included adjustment for pre-injury EQ-5D status and time between injury and when information was obtained from participants.

**Results:**

Substantial portions of participants continued to have adverse outcomes approximately three months after their injury. Key pervasive factors predicting adverse outcomes were: being female, prior chronic illness, injuries to multiple body regions, being hospitalized for injury, self-perceived threat to life, and difficulty accessing health services.

**Conclusion:**

Future injury outcome studies should include participants whose injuries are considered 'minor', as judged by acute health service utilization, and also consider a wider range of potential predictors of adverse outcomes.

## Background

Studies of outcomes following specific injury types have provided some useful insights into the potential predictors of functional outcomes following injury such as: injury characteristics, health service factors, depression, stress, recovery expectations and employment characteristics. However, conclusions have been constrained by: inclusion of a narrow range of predictor or outcome variables, collection of limited pre-injury characteristics, poor follow-up rates, and selective recruitment or follow-up of predominantly high threat to life injuries [1]. The last point is of particular importance since some injuries that are minor, in terms of threat to life, can result in serious functional limitations. Moreover, in relation to the overall burden imposed by injury, such low threat to life injuries are considerably more numerous than those injuries that require acute hospital inpatient treatment.

This paper aims to identify the role of pre-injury socio-demographic and health characteristics, injury and injury-related healthcare in determining short-term functional outcomes following a wide range of injury types. The underlying hypothesis of this investigation is that, aside from the nature of injury, a range of other factors are important in explaining short-term functional outcomes.

## Methods

This paper uses data from the first interview with participants in the Prospective Outcomes of Injury Study (POIS). POIS is a prospective cohort study of 2856 injured persons who will be interviewed four times over a 24-month period. The study received ethical approval from the New Zealand Health and Disability Multi-region Ethics Committee (MEC/07/07/093). The protocol for this study has been described elsewhere [2]; a brief overview is provided below.

### Study Population

The study population was New Zealand residents aged 18 to 64 years (inclusive), referred to the Accident Compensation Corporation (ACC) for case co-ordination and/or management for an acute (rather than gradual onset) injury. Those whose injury was the result of self-harm or sexual assault were excluded.

The ACC manages New Zealand's 'no-fault' comprehensive injury cover for all New Zealand residents and visitors [3]. Injured people can apply for assistance, no matter how they became injured, or who was at fault. The assistance can include a wide range of services from payment for treatment and equipment, to help with income if the injured person can no longer work. Because of the wide range of help available from ACC after an injury, people cannot sue for personal injury in New Zealand, except for exemplary damages.

About 7% of the 1.75 million new injury claims registered with ACC each year potentially require compensation and/or support for returning to independence (e.g. income support, home support or assistance with returning to work). These are referred to as entitlement claims [4]. Participants in our study were recruited from the population of injured people referred to ACC for some form of injury entitlement.

### Cohort Recruitment

Cohort members resided in one of five ACC regions of New Zealand - Auckland, Manukau City, Gisborne, Otago, and Southland. Each month, potential participants were selected from new ACC claimants. ACC then sent, on our behalf, a letter of invitation and our Study Information Sheet to these selected potential participants. We then independently contacted these potential participants and collected informed consent before any interviews occurred.

### Data Collection

Trained interviewers collected information on the variables of interest from participants. Most (89%) were interviewed by telephone; postal surveys were completed by 328 (11%) and face-to-face interviews were conducted for less than 1%. All participants received a $10 voucher in acknowledgement of their involvement. Between December 2007 and August 2009, 2856 participants were recruited. The median time to interview was 3.2 months post-injury (25^th ^percentile: 2.5 months, and 75^th ^percentile: 4.2 months).

### Outcome Measures

The EQ-5D was selected to assess functional outcome [5]. The EQ-5D is a general measure of health status which defines health along five dimensions (mobility, self-care, usual activities, pain or discomfort, anxiety or depression) [6]. Each dimension contains three response options indicating no problems, some problems or extreme problems with the specified dimension.

To capture the consequences of head injury, we added a question, as others have previously done, on cognitive ability using the same format as the five EQ-5D dimensions [7]:

*"The next statements relate to intellectual activities such as remembering, concentrating, thinking and solving day to day problems"*.

In addition to asking participants to describe functional outcomes after injury, we asked them to characterize their pre-injury functional status for each dimension.

### Explanatory Variables

Our review of the literature suggested a range of potential pre-injury and injury-related explanatory variables [2]. We grouped our explanatory variables into: pre-injury socio-demographic, pre-injury health and disability, and injury and post-injury healthcare characteristics. Questions relating to pre-injury characteristics made it clear that we wished the participants to report on the period immediately prior to injury.

### Pre-injury socio-demographic characteristics

Questions relating to gender and age replicated those in the New Zealand Census 2006 [8]. For the purposes of determining living arrangements, we used the Census question which seeks to elicit the number of people living in the same household and their relationship with the respondent [8]. We assessed the highest educational qualification using the two questions from the Census [8]. The first of these determines the highest school qualification and the second any other qualification that took three months, or more, to obtain. Participants were asked about their involvement in paid work. Participants who responded that they were working full-time (≥ 30 hours per week) or part-time (< 30 hours per week) were classified as working for pay [9]. Financial status was assessed using a question from the Statistics New Zealand Household Economic Survey 2006 which asked people to rate the adequacy of their total household income to meet their everyday needs for things such as accommodation, food, clothing and other daily necessities on a four-point scale ranging from 'not enough' to 'more than enough' [10]. Those responding 'not enough' were classified as having insufficient financial standing.

### Pre-injury health and disability characteristics

To ascertain pre-injury disability, questions from the New Zealand Census 2006 were modified, by adding the bolded words below [8]:

"**Before your injury**, did a health problem or condition you have (lasting 6 months or more) cause you difficulty with, or stop you doing:

Everyday activities that people your age do?

Communicating, mixing with others or socializing?

Or any other activity that people your age can usually do?

No difficulty with any of these?"

If people responded positively to any of the first three questions they were classified as having a pre-injury disability.

Prior chronic illness was assessed using a modified instrument developed for the New Zealand Health Survey 2006/2007 [11]. Participants were asked if they had been told by a doctor that they had any of 22 specified chronic illnesses or diseases such as asthma, cancer, diabetes, depression or anxiety that had lasted, or was expected to last, for more than six months. Overall health was assessed by asking participants to rate their pre-injury health, in general, on a five-point scale ('Excellent', 'Very Good', 'Good', 'Fair' or 'Poor') [12]. Participants were asked to report their height and weight from which we derived their Body Mass Index (BMI); underweight (BMI < 20), normal (20-24.9), overweight (25-29.9) and obese (≥ 30) [13].

Optimism was measured by a single question from the Life Orientation Test asking for agreement or disagreement on a five-point scale with overall expectations of 'more good things happening to them than bad' [14]. Participants who strongly agreed or agreed were compared with the rest. The self-efficacy measure was based on the General Self-Efficacy Scale, a 10-item psychometric scale that is designed to assess optimistic self-beliefs to cope with a variety of difficult demands in life [15]. Response categories used for the items were: 'strongly disagree', 'disagree', 'neutral/mixed', 'agree', and 'strongly agree' scored 0 to 4 respectively. We defined poor self-efficacy as a score of ≤ 25 out of a maximum possible score of 40. Indication of a major depressive episode was assessed by using two DSM-III screening questions for depressed mood or loss of interest or pleasure in daily activities consistently for at least a two-week period in the 12 months before injury [16]. Comfort in faith and spiritual belief was assessed using a single question from the FACIT-Sp (permission to use the item was granted by http://www.facit.org), which had five response options ranging from 'Not at all' to 'Very much' [17]:

*"I find comfort in my faith or spiritual beliefs"*.

Smoking behaviour was determined using a question directly from the New Zealand Census 2006 [8]:

"Do you smoke cigarettes regularly (that is, one or more a day)?"

The condensed form of the Alcohol Use Disorders Identification Test (AUDIT-C) was used to identify participants who had potentially hazardous drinking patterns or active alcohol use disorders (including alcohol abuse or dependence). AUDIT-C scores range between 0 and 12 [18]. A score of 4 or more was considered indicative of hazardous drinking for men; a score of 3 or more hazardous for women. Participants were also asked:

"In the year before your injury, how often did you use marijuana or cannabis?"

The response categories were: 'Never', 'Monthly or less', '2-4 times a month', '2-3 times a week', '4 or more times a week'. A similar question, with the same response categories, was asked for other recreational drugs:

"In the year before your injury, how often did you use any other recreational drugs such as P, speed, ecstasy, LSD, or cocaine?"

In both these questions participants who responded other than 'Never' were categorized as users.

Frequency of pre-injury physical activity was evaluated by asking participants the number of times in the seven-day period prior to injury they had engaged in either 30 minutes of moderate activity (including brisk walking) or 15 minutes of vigorous activity that made them breathe a lot harder than usual ('huff and puff') [19]. Participants were categorized as physically active if they undertook either activity for at least five days in that week.

### Injury and healthcare characteristics

Participants were asked to identify which body part(s) had been injured and also the type(s) of injury (such as fracture, sprain) sustained. This information was classified according to body regions and nature of injury based on a modified version of the Barell Matrix [20]. To determine the intent of the injury event, participants were asked if their injury was due to an accident or physical assault.

Participants were asked whether they had been admitted to hospital for one day only (no nights) or for one night or more as a result of their injury. Participants were also asked for their assessment of the seriousness of the injury in terms of threat to life and threat of disability. The questions were:

*"At the time, did you feel the injury was a threat to your life?" *and

"A threat of severe longer-term disability to you?"

Response categories were 'Yes', 'Maybe/possibly', or 'No'. For the multivariate analyses, the first two categories were grouped together.

Access to healthcare services was ascertained by asking the following open-ended question:

"Did you have trouble getting to or contacting health services?"

Participants were classified as having had trouble accessing health services if they responded 'Yes' or 'Mixed' rather than 'No'.

### Statistical Analysis

Bivariate analyses were completed first to enable associations between 'Any problems' (combining 'Some' and 'Extreme' problem responses) with the six outcomes of interest (the five dimensions of the EQ-5D, and cognitive ability) and the independent measures to be assessed.

For the six binary outcome measures, separate multivariate logistic regression models were built to predict responses, while adjusting for the same respective pre-injury characteristic. For example, when mobility was the outcome measure of interest, pre-injury mobility status was included in the model. As mentioned, the time period between injury and interview varied between participants and was also significantly associated with a range of outcome measures. Therefore, time since injury was adjusted for in all multivariate models. All variables described above were included in the model initially. As it was desired to have a consistent set of explanatory variables in all six functional outcome models, the contribution of a variable to the models was considered across all six of the models. Pre-injury health status, age, gender and injury body region and type were considered important to retain in the multivariate models based on previous research. Other explanatory variables were removed from the models if all six p-values for a particular variable were ≥ 0.1. Models for the six binary outcome measures were re-estimated on the remaining subset of variables. Variables were removed from the models one at a time until all variables had p ≤ 0.05 for at least one of the six outcome measures. The decision as to which order variables should be removed was based on assessing the p-values across all six models with the variable having the highest p-values being removed first.

Multivariate logistic regression models for the six binary outcome variables were also fitted without forcing a consistent set of explanatory variables. All variables described above were included in each of the initial dimension-specific models. Similarly time since injury, pre-injury health status, age, gender, injury body region and type were forced to be included in the final six dimension-specific models. Model building was completed independently for each outcome with variables removed one at a time until all variables had p ≤ 0.05.

Stata 11.1 was used for the analysis [21].

## Results

The prevalence of 'any problems' pre-injury ranged from 2% to 11% (Table 1). In all cases the prevalence was substantially elevated following injury (3 to 12 fold increase). The post-injury prevalences of pain or discomfort (69%), usual activities (54%) and mobility (41%) were particularly high.

**Table 1:** Prevalence of any problems before and after injury in EQ-5D Dimensions & Cognitive Ability (N = 2856)

	EQ-5D Dimensions											
			
	Mobility		Self-Care		Usual Activities		Pain, Discomfort		Anxiety, Depression		Cognitive	
	
	%	(95%CI)	%	(95%CI)	%	(95%CI)	%	(95%CI)	%	(95%CI)	%	(95%CI)
**Pre-injury**	6	(5, 7)	2	(2, 3)	6	(5, 6)	11	(10, 12)	6	(5, 7)	5	(4, 6)

**3 months after injury**	41	(39, 43)	24	(22, 25)	54	(52, 56)	69	(67, 71)	23	(21, 24)	15	(13, 16)

### Bivariate Analyses

The bivariate results for the socio-demographic characteristics (Table 2) show that, with the exception of living arrangements, most of the characteristics are associated with at least one of the six outcomes. Of note are the findings that females had higher prevalences of problems for all outcomes, and that as age increased so did the prevalence of problems with mobility, self-care, usual activities and pain or discomfort.

**Table 2:** Prevalence of any problems in EQ-5D Dimensions & Cognitive Ability by pre-injury socio-demographic characteristics

			EQ-5D Dimensions											
			
			Mobility		Self-Care		Usual Activities		Pain, Discomfort		Anxiety, Depression		Cognitive	
	
	N	%	%	(95% CI)	%	(95% CI)	%	(95% CI)	%	(95% CI)	%	(95% CI)	%	(95% CI)
**Gender**														

Male	1,753	61	38	(36, 41)	22	(20, 24)	51	(48, 53)	66	(64, 69)	21	(19, 23)	13	(12, 15)

Female	1,103	39	46	(43, 48)	26	(24, 29)	59	(56, 62)	74	(71, 76)	25	(22, 27)	17	(14, 19)

**Age**														

18-24 yrs	396	14	31	(27, 36)	19	(15, 23)	49	(44, 54)	59	(54, 64)	21	(17, 26)	14	(11, 18)

25-44 yrs	1,223	43	39	(37, 42)	22	(19, 24)	54	(51, 57)	71	(68, 73)	22	(20, 25)	15	(13, 18)

45-64 yrs	1,237	43	46	(43, 49)	27	(25, 30)	55	(53, 58)	71	(68, 73)	23	(21, 26)	14	(12, 16)

**Living arrangements**														

Alone	272	10	40	(34, 46)	25	(20, 30)	53	(47, 59)	68	(62, 73)	24	(19, 30)	18	(14, 23)

Living with non-family	260	9	39	(33, 45)	20	(16, 26)	55	(49, 61)	63	(57, 69)	24	(19, 30)	16	(12, 22)

Living with partner/family/relative	2,308	81	41	(39, 43)	24	(22, 26)	54	(52, 56)	70	(68, 72)	22	(20, 24)	14	(12, 15)

**Highest educational qualificaton**														

None	428	15	42	(38, 47)	27	(23, 31)	55	(50, 60)	70	(65, 74)	28	(23, 32)	18	(14, 22)

Secondary school	683	24	40	(36, 44)	19	(16, 22)	51	(47, 55)	66	(62, 69)	21	(18, 24)	12	(10, 15)

Post-secondary school	1,676	59	41	(39, 44)	25	(23, 27)	55	(53, 58)	71	(68, 73)	22	(20, 24)	14	(13, 16)

**Working for pay**														

No	229	8	53	(47, 60)	28	(22, 34)	61	(54, 67)	73	(67, 79)	30	(24, 37)	22	(17, 28)

Yes	2,626	92	40	(38, 42)	23	(22, 25)	53	(51, 55)	69	(67, 71)	22	(20, 23)	14	(12, 15)

**Financial status**														

Insufficient	270	9	43	(37, 49)	31	(25, 37)	59	(53, 65)	74	(69, 80)	34	(28, 40)	21	(16, 26)

Sufficient	2,553	89	41	(39, 43)	23	(21, 25)	53	(51, 55)	69	(67, 70)	21	(20, 23)	14	(13, 15)

The results for the pre-injury health and disability characteristics (Table 3) show that disability, two or more chronic illnesses, and depressed episode in the last 12 months are associated with all of the outcomes. The remaining variables have either no association or were associated with only one or two of the six outcomes.

**Table 3:** Prevalence of any problems in EQ-5D Dimensions & Cognitive Ability by pre-injury health and disability characteristics

			EQ-5D Dimensions											
			
			Mobility		Self-Care		Usual Activities		Pain, Discomfort		Anxiety, Depression		Cognitive	
	
	N	%	%	(95% CI)	%	(95% CI)	%	(95% CI)	%	(95% CI)	%	(95% CI)	%	(95% CI)
**Disability**														

No	2370	83	39	(37, 41)	22	(21, 24)	52	(50, 54)	67	(65, 69)	21	(20, 23)	13	(11, 14)

Yes	434	15	54	(49, 58)	31	(26, 35)	66	(61, 70)	81	(77, 85)	28	(24, 33)	23	(19, 28)

**Chronic illness**														

None	1,432	50	38	(36, 41)	22	(20, 24)	51	(48, 54)	66	(64, 69)	19	(17, 21)	12	(10, 13)

One	759	27	39	(35, 42)	23	(20, 26)	52	(49, 56)	68	(64, 71)	21	(18, 24)	13	(11, 15)

Two or more	567	20	51	(47, 56)	30	(26, 33)	63	(59, 67)	79	(75, 82)	34	(30, 38)	24	(21, 28)

**Overall health**														

Fair/Poor	160	6	45	(37, 53)	22	(16, 29)	56	(48, 64)	76	(69, 83)	28	(21, 35)	30	(23, 38)

Good	723	25	42	(39, 46)	24	(21, 28)	56	(52, 60)	69	(65, 72)	26	(23, 30)	18	(15, 21)

Very Good/Excellent	1,966	69	40	(38, 43)	24	(22, 26)	53	(51, 55)	69	(67, 71)	21	(19, 23)	12	(11, 13)

**Body Mass Index**														

Underweight	33	1	45	(28, 64)	9	(2, 24)	45	(28, 64)	79	(61, 91)	15	(5, 32)	21	(9, 39)

Normal	973	34	38	(34, 41)	22	(19, 25)	54	(51, 57)	69	(66, 72)	23	(20, 26)	13	(11, 16)

Overweight	1,040	36	40	(37, 43)	24	(21, 26)	52	(49, 55)	68	(65, 71)	23	(20, 25)	13	(11, 16)

Obese	684	24	47	(43, 51)	26	(23, 30)	58	(54, 61)	71	(67, 74)	21	(18, 24)	16	(14, 19)

**Optimistic**														

No	339	12	46	(41, 51)	27	(23, 33)	56	(51, 62)	73	(68, 78)	30	(25, 35)	18	(14, 23)

Yes	2,475	87	41	(39, 42)	24	(22, 25)	54	(52, 56)	69	(67, 71)	22	(20, 23)	14	(13, 16)

**General self-efficacy**														

Poor	278	10	45	(39, 51)	29	(24, 34)	58	(52, 64)	72	(66, 77)	32	(26, 37)	23	(18, 29)

Good	2,547	89	41	(39, 43)	23	(22, 25)	54	(52, 56)	69	(67, 71)	22	(20, 23)	14	(12, 15)

**Depressed**														

No	2,138	75	40	(37, 42)	23	(21, 24)	52	(50, 54)	68	(66, 70)	18	(17, 20)	12	(11, 13)

Yes	714	25	46	(42, 50)	27	(24, 31)	60	(56, 64)	73	(70, 77)	35	(32, 39)	22	(19, 26)

**Comfort in faith or spiritual beliefs**														

Not at all	868	30	39	(36, 42)	21	(18, 24)	53	(50, 57)	66	(63, 69)	20	(17, 23)	12	(10, 14)

A little bit/Somewhat	893	31	42	(39, 45)	23	(20, 25)	54	(50, 57)	71	(68, 74)	24	(21, 27)	15	(13, 18)

Quite a bit/Very much	968	34	43	(40, 46)	28	(25, 31)	55	(52, 58)	69	(66, 72)	24	(21, 27)	16	(14, 19)

**Smoke regularly**														

No	1,990	70	42	(40, 44)	23	(21, 25)	54	(52, 56)	69	(66, 71)	21	(19, 23)	13	(12, 15)

Yes	856	30	39	(36, 42)	26	(23, 29)	54	(51, 57)	71	(68, 74)	26	(23, 29)	17	(15, 20)

**Hazardous alcohol use**														

No	964	34	43	(40, 46)	25	(22, 28)	53	(50, 56)	68	(65, 71)	24	(21, 27)	15	(13, 18)

Yes	1,860	65	40	(38, 43)	23	(21, 25)	54	(52, 57)	70	(68, 72)	22	(20, 24)	14	(13, 16)

**Illicit Drug use**														

No	2,333	82	42	(40, 44)	24	(23, 26)	54	(52, 56)	69	(67, 71)	22	(20, 24)	14	(12, 15)

Yes	513	18	35	(31, 40)	21	(17, 25)	54	(49, 58)	70	(65, 73)	25	(21, 29)	18	(15, 21)

**Physically active**														

No	1,254	44	40	(37, 43)	22	(20, 25)	52	(49, 55)	68	(65, 70)	20	(18, 23)	13	(12, 15)

Yes	1,541	54	42	(39, 44)	25	(23, 27)	56	(53, 58)	70	(68, 73)	24	(22, 26)	15	(13, 17)

Among the injury and healthcare characteristics (Table 4), those with lower extremity injuries had a high prevalence of mobility problems; pain or discomfort was more likely to be associated with spine and back injuries and injuries to multiple regions. In terms of the nature of injury, notable findings were that those with sprains and strains were more likely to experience mobility problems and those with concussion were more likely to experience problems with usual activities and cognition. Being admitted to hospital for injury, perceived threat of disability and trouble accessing health services were associated with all six outcomes. Injuries resulting from assault were associated with anxiety and depression and cognitive problems.

**Table 4:** Prevalence of any problems in EQ-5D Dimensions & Cognitive Ability by injury and healthcare characteristics

			EQ-5D Dimensions											
			
			Mobility		Self-Care		Usual Activities		Pain, Discomfort		Anxiety, Depression		Cognitive	
	
	N	%	%	(95% CI)	%	(95% CI)	%	(95% CI)	%	(95% CI)	%	(95% CI)	%	(95% CI)
**Body region injured**														

Lower Extremity	1081	38	64	(61, 67)	20	(17, 22)	54	(51, 57)	70	(67, 73)	20	(18, 22)	9	(7, 11)

Upper Extremity	783	27	9	(7, 11)	27	(24, 31)	48	(44, 51)	66	(63, 69)	19	(17, 22)	11	(9, 13)

Head & Neck	125	4	23	(16, 31)	10	(6, 17)	52	(42, 61)	48	(39, 58)	33	(25, 42)	41	(32, 50)

Spine & Back	269	9	54	(48, 60)	30	(25, 36)	61	(55, 67)	74	(69, 80)	24	(19, 29)	18	(14, 23)

Torso	69	2	22	(13, 33)	17	(9, 28)	35	(24, 47)	58	(46, 70)	10	(4, 20)	6	(2, 14)

Multiple regions	529	19	42	(38, 47)	28	(24, 32)	63	(59, 67)	76	(72, 79)	31	(27, 35)	25	(21, 28)

**Nature of injury**														

Fractures	513	18	36	(32, 40)	24	(20, 28)	51	(47, 56)	66	(62, 70)	20	(17, 24)	9	(7, 12)

Sprains & Strains	729	26	50	(47, 54)	23	(20, 26)	54	(51, 58)	73	(69, 76)	21	(18, 24)	10	(8, 13)

Concussion	22	1	23	(8, 45)	5	(0, 23)	68	(45, 86)	59	(36, 79)	32	(14, 55)	46	(24, 68)

Open Wounds/Amputations	132	5	14	(9, 22)	17	(11, 24)	36	(28, 45)	54	(45, 63)	17	(11, 24)	8	(4, 14)

Contusions/Superficial	70	2	39	(27, 51)	17	(9, 28)	39	(27, 51)	57	(45, 69)	21	(13, 33)	11	(5, 21)

Other single injury type	279	10	37	(31, 43)	26	(21, 32)	49	(43, 55)	64	(58, 70)	22	(17, 27)	13	(10, 18)

Multiple injury types	1111	39	42	(39, 45)	25	(23, 28)	59	(56, 62)	72	(70, 75)	26	(23, 28)	20	(18, 23)

**Intent of injury event**														

Accidental	2,729	95	41	(40, 43)	24	(22, 25)	53	(52, 55)	69	(68, 71)	22	(20, 23)	13	(12, 15)

Assault	113	4	33	(24, 42)	25	(17, 34)	62	(52, 71)	67	(58, 76)	40	(31, 49)	39	(30, 49)

**Admitted to hospital**														

Yes	871	31	48	(44, 51)	30	(27, 33)	62	(59, 66)	73	(70, 76)	28	(25, 31)	19	(16, 22)

No	1,970	69	38	(36, 41)	21	(19, 23)	50	(48, 52)	68	(66, 70)	20	(19, 22)	13	(11, 14)

**Self-perceived threat to life**														

Yes	247	9	53	(47, 59)	37	(31, 43)	64	(58, 70)	74	(68, 79)	43	(37, 49)	32	(26, 38)

Maybe/Possibly	93	3	48	(38, 59)	23	(15, 32)	71	(61, 80)	78	(69, 86)	38	(28, 48)	31	(22, 42)

No	2,469	86	40	(38, 42)	22	(21, 24)	52	(50, 54)	68	(66, 70)	19	(18, 21)	12	(10, 13)

**Self-perceived threat of disability**														

Yes	799	28	50	(46, 53)	31	(27, 34)	63	(60, 67)	79	(76, 82)	32	(29, 36)	19	(16, 22)

Maybe/Possibly	368	13	42	(37, 47)	22	(17, 26)	56	(50, 61)	72	(67, 77)	20	(16, 24)	13	(10, 17)

No	1,630	57	36	(34, 39)	20	(18, 22)	48	(46, 51)	63	(61, 65)	18	(16, 20)	12	(10, 13)

**Access to healthcare services**														

No trouble	2,540	89	40	(38, 42)	23	(21, 24)	52	(50, 54)	68	(66, 70)	21	(19, 23)	13	(12, 15)

Trouble	288	10	52	(46, 58)	33	(28, 39)	70	(64, 75)	81	(76, 85)	36	(30, 41)	24	(19, 30)

### Multivariate Analyses

Table 5 presents the estimates obtained when a consistent set of explanatory and confounder variables were included in the six models. Comparison of these estimates with those obtained from the six dimension-specific models indicated no substantive differences, therefore the dimension-specific models are not presented.

**Table 5:** Multivariate analysis of factors associated with problems in EQ-5D Dimensions & Cognitive Ability

	EQ-5D Dimensions											
	
	Mobility		Self-Care		Usual Activities		Pain, Discomfort		Anxiety, Depression		Cognitive	
	
	N = 2460		N = 2461		N = 2460		N = 2456		N = 2459		N = 2458	
	
	OR	95% CI	OR	95% CI	OR	95% CI	OR	95% CI	OR	95% CI	OR	95% CI
**Socio-demographic**												

Female	1.34	(1.09, 1.65)	1.40	(1.14, 1.73)	1.53	(1.28, 1.84)	1.62	(1.32, 1.97)	1.23	(0.98, 1.53)	1.63	(1.21, 2.20)

Age (yrs)												

*18-24*	1.00	ref	1.00	ref	1.00	ref	1.00	ref	1.00	ref	1.00	ref

*25-44*	1.17	(0.86, 1.61)	1.09	(0.79, 1.52)	1.18	(0.91, 1.54)	1.74	(1.32, 2.28)	1.13	(0.81, 1.59)	1.13	(0.71, 1.79)

*45-64*	1.61	(1.16, 2.21)	1.60	(1.15, 2.23)	1.25	(0.96, 1.64)	1.74	(1.31, 2.30)	1.38	(0.98, 1.96)	0.96	(0.59, 1.55)

Insufficient money	1.25	(0.89, 1.77)	1.33	(0.96, 1.84)	1.26	(0.94, 1.70)	1.34	(0.96, 1.87)	1.70	(1.22, 2.37)	0.97	(0.60, 1.56)

**Health and disability**												

Disability	1.36	(1.01, 1.82)	1.05	(0.79, 1.40)	1.17	(0.90, 1.52)	1.46	(1.08, 1.99)	0.79	(0.58, 1.07)	0.81	(0.53, 1.21)

Two or more chronic illnesses	1.47	(1.12, 1.92)	1.18	(0.90, 1.53)	1.27	(1.00, 1.62)	1.52	(1.16, 2.00)	1.62	(1.24, 2.11)	1.79	(1.26, 2.54)

Fair/Poor overall health	0.92	(0.73, 1.15)	0.78	(0.62, 0.98)	0.90	(0.73, 1.09)	0.81	(0.66, 1.01)	1.10	(0.87, 1.40)	1.42	(1.04, 1.95)

Obese	1.23	(0.98, 1.54)	1.26	(1.01, 1.58)	1.21	(0.99, 1.48)	1.04	(0.84, 1.29)	0.86	(0.67, 1.10)	1.26	(0.92, 1.73)

Depressed	1.06	(0.83, 1.35)	1.16	(0.91, 1.47)	1.14	(0.92, 1.42)	0.96	(0.76, 1.21)	1.57	(1.23, 2.01)	1.14	(0.81, 1.59)

Smoke regularly	1.13	(0.90, 1.41)	1.21	(0.97, 1.50)	1.02	(0.84, 1.24)	1.24	(1.01, 1.54)	1.19	(0.95, 1.50)	1.26	(0.93, 1.73)

Hazardous alcohol use	1.03	(0.84, 1.27)	0.96	(0.78, 1.19)	1.22	(1.01, 1.46)	1.24	(1.02, 1.51)	0.85	(0.68, 1.06)	0.77	(0.57, 1.05)

Physically inactive	0.89	(0.73, 1.08)	0.82	(0.67, 1.00)	0.83	(0.70, 0.99)	0.82	(0.68, 0.99)	0.73	(0.59, 0.90)	0.68	(0.51, 0.91)

**Injury and healthcare**												

Body region injured												

*Lower Extremity*	3.17	(2.40, 4.18)	0.73	(0.54, 0.99)	0.75	(0.57, 0.98)	0.79	(0.59, 1.06)	0.68	(0.50, 0.93)	0.42	(0.28, 0.62)

*Upper Extremity*	0.15	(0.10, 0.21)	1.27	(0.93, 1.73)	0.70	(0.53, 0.93)	0.78	(0.57, 1.06)	0.64	(0.46, 0.89)	0.46	(0.30, 0.71)

*Head & Neck*	0.41	(0.23, 0.73)	0.41	(0.20, 0.80)	0.53	(0.32, 0.87)	0.29	(0.17, 0.48)	0.95	(0.56, 1.63)	2.26	(1.27, 4.01)

*Spine & Back*	1.74	(1.19, 2.53)	1.30	(0.87, 1.95)	1.12	(0.78, 1.62)	0.84	(0.56, 1.26)	0.68	(0.44, 1.04)	1.02	(0.60, 1.73)

*Torso*	0.38	(0.19, 0.77)	0.72	(0.35, 1.47)	0.37	(0.20, 0.68)	0.54	(0.30, 0.99)	0.27	(0.11, 0.68)	0.11	(0.02, 0.57)

*Multiple regions*	1.00	ref	1.00	ref	1.00	ref	1.00	ref	1.00	ref	1.00	ref

Nature of injury												

*Fractures*	0.87	(0.64, 1.17)	1.18	(0.88, 1.58)	0.92	(0.71, 1.18)	0.98	(0.75, 1.28)	1.03	(0.75, 1.41)	0.66	(0.42, 1.04)

*Sprains & Strains*	1.16	(0.89, 1.50)	1.21	(0.92, 1.59)	1.08	(0.86, 1.36)	1.19	(0.92, 1.53)	1.04	(0.78, 1.38)	0.65	(0.43, 0.98)

*Concussion*	1.06	(0.30, 3.76)	0.48	(0.06, 3.97)	2.09	(0.67, 6.49)	1.07	(0.35, 3.29)	1.03	(0.31, 3.39)	1.89	(0.61, 5.85)

*Open Wounds/Amputations*	0.40	(0.21, 0.75)	0.60	(0.35, 1.03)	0.45	(0.29, 0.69)	0.49	(0.32, 0.75)	0.65	(0.36, 1.15)	0.52	(0.23, 1.20)

*Contusions/Superficial*	0.69	(0.36, 1.33)	0.62	(0.29, 1.32)	0.46	(0.25, 0.83)	0.55	(0.31, 0.99)	0.82	(0.39, 1.71)	0.36	(0.11, 1.18)

*Other single injury type*	1.03	(0.71, 1.50)	1.16	(0.81, 1.68)	0.74	(0.54, 1.02)	0.72	(0.52, 1.02)	1.17	(0.80, 1.73)	0.69	(0.41, 1.17)

*Multiple injury types*	1.00	ref	1.00	ref	1.00	ref	1.00	ref	1.00	ref	1.00	ref

Assaultive intent	1.09	(0.46, 1.95)	1.36	(0.79, 2.33)	1.38	(0.84, 2.27)	1.19	(0.70, 2.02)	1.71	(1.01, 2.88)	2.74	(1.56, 4.82)

Admitted to hospital	1.76	(1.41, 2.20)	1.71	(1.37, 2.13)	1.84	(1.51, 2.23)	1.42	(1.15, 1.75)	1.38	(1.10, 1.74)	1.53	(1.12, 2.08)

Self-perceived threat to life	1.37	(1.00, 1.88)	1.43	(1.05, 1.95)	1.28	(0.95, 1.73)	1.06	(0.76, 1.48)	1.83	(1.35, 2.50)	2.80	(1.93, 4.07)

Self-perceived threat of disability	1.41	(1.15, 1.73)	1.31	(1.06, 1.62)	1.34	(1.12, 1.60)	1.87	(1.53, 2.29)	1.41	(1.13, 1.76)	0.81	(0.60, 1.11)

Trouble accessing healthcare services	1.79	(1.30, 2.47)	1.52	(1.12, 2.07)	1.88	(1.40, 2.52)	1.72	(1.22, 2.41)	1.77	(1.30, 2.42)	2.17	(1.47, 3.20)

Diagnostic testing of the final models presented in Table 5 indicated goodness of fit was acceptable with p-values from the Hosmer-Lemeshow test ranging from 0.25 to 0.63. The models also had good accuracy in correctly discriminating if participants had functional outcome problems with areas under curves of 0.84 and 0.82 for cognitive ability and mobility, respectively; the remainder ranged between 0.69 and 0.73.

Among the socio-demographic variables, the most notable findings were: 1) females had elevated odds for all the outcomes except anxiety or depression, and 2) those 45-64 years of age had elevated risk of problems with mobility, self-care, and pain or discomfort (Table 5).

Among the pre-injury health and disability characteristics, notable findings were: 1) two or more prior chronic illnesses was related to elevated odds for all outcomes, with the exception of self-care, 2) disability was related to elevated risk of mobility, and pain or discomfort, 3) reports of feeling depressed in the 12 months prior to injury were associated with a higher risk of anxiety or depression, 4) with the exception of mobility, being physically inactive appeared to reduce the risk of problems, particularly cognitive problems.

Among the injury and healthcare characteristics, findings of note were: 1) participants with injuries to multiple regions were typically more likely to have adverse scores than those with an injury to one region only (exceptions to this were the associations between lower extremity and spine and back injuries and mobility problems, and injuries to the head and neck and cognitive problems), 2) when injury resulted from assault, participants were at increased risk of problems with cognitive ability and anxiety or depression, 3) being admitted to hospital increased the odds of problems with all outcomes, most notably for mobility, self-care and usual activities, 4) perceived threat to life was strongly associated with anxiety or depression and cognitive problems, 5) perceived threat of disability was associated with all outcomes except cognitive problems, the strongest relationship being with pain or discomfort, and 6) trouble accessing health services was strongly associated with all outcomes.

## Discussion

Previous injury outcome studies tend to have either been based on trauma patients (e.g. [22,23]) or hospital in- and out-patients [24,25]. Our study population was considerably wider in scope as it included persons who had been injured and had sought assistance from primary or secondary healthcare services. This would include, for example, someone who was treated solely by a general practitioner in the acute phase and was eligible for time off work to assist in recovery. Our findings are thus not directly comparable with previous studies. The rationale for selecting our study population was that there are many injuries that do not result in acute hospital service utilization but nevertheless may incur significant adverse outcomes. The findings presented here bear that out. For example, 31% of participants were admitted to hospital for the treatment of their injury. Of these, 62% had problems performing their usual activities. However, 50% of the non-admitted participants also had problems performing their usual activities (Table 4). Nevertheless, as anticipated, being admitted to hospital was associated with an independent and detrimental effect on all outcomes (Table 5).

While it seems reasonable to assume that being admitted to hospital would affect many victims' perceived threat to life or threat of disability, it is noteworthy that these perceptions both had independent effects. Holbrook and others have previously reported that perceived threat to life is strongly and independently associated with post-traumatic stress disorder [26]. Our findings for anxiety or depression are consistent with that study (Table 5). It is possible that perceived threat to life may not be highly correlated with empirically derived measures of threat to life given the average lay person's knowledge of physiological or anatomical risk. Once we obtain hospital discharge data related to the participants who were admitted to hospital for treatment of their injury, we will seek to determine whether the observed effect remains after controlling for an empirically derived estimate of threat to life. A recent Swiss-based study reported that patient appraisal of injury severity was predictive of time off work following life threatening injuries but that the objective measure of severity was not [27].

Perceived threat of disability was strongly related to problems with pain or discomfort. While relationships were observed for several of the other outcomes, they were weak. We are unaware of any previous research that has investigated this relationship.

Trouble accessing healthcare services predicted poorer outcomes across all measures. A review of healthcare service use among injured and non-injured populations found a dearth of published studies reporting health service utilization outcomes [28]. Similarly, we have been unable to find studies reporting difficulties of access to healthcare services in relation to functional outcomes after injury. However, relationships between timeliness of access to healthcare services and survival following injury have been demonstrated [29]. Research investigating death following injury using American trauma registry data found people with no insurance were at increased risk of death [30]. They hypothesized that a reason for this may be that the uninsured had poorer access to healthcare services, be this through delay or different types of services being provided. Although this finding was consistent for younger participants (18-30 years), a limitation in their study was an inability to adjust for co-morbidity. We found that trouble accessing healthcare services predicted poorer functional outcomes, when adjusting for co-morbidity and a range of other factors.

The estimates for the specific injury categories excluded those same injuries when they occurred along with others (these were instead categorized as 'multiple'). Other investigators have dealt with this issue by identifying the principal or most severe injury [24,25]. Identifying the principal or most severe injury is typically done by reference to the degree of anatomical damage. Aside from the fact that the information provided by participants in our study would have been inadequate for that purpose, adopting such an approach would have seriously compromised one of the primary intentions of our study, namely to determine to what degree injuries resulting in relatively minor anatomical injury result in adverse outcome. Holtslag and others addressed this issue by producing estimates for each specific injury versus those not having that specific injury (i.e. all other injuries) [23]. That is, if a patient had two or more specific injuries they were included in all relevant groups. They addressed the issue of the independent impact of multiple injuries by including in their model the Injury Severity Score, an anatomical scale for multiple injury [31]. For the reasons outlined above that was not an option for us. We nevertheless felt it important to determine the impact of injuries to multiple regions hence the strategy we adopted.

Similar caution needs to be exercised in interpreting our findings for the nature of injury. Because we used participants' descriptions of injury we could not reliably determine whether a reported second injury was in fact one normally associated with the first injury and, as such, not usually counted. All such cases were treated as multiple injury types.

Our finding that assault predicted anxiety or depression is consistent with previous work that has shown a relationship between assault and post-traumatic stress disorder [22].

In contrast to the injury factors, few of the socio-demographic factors remained in the final model. Contrary to what we hypothesized, living arrangements, educational qualifications, and whether one was working for pay were not independently related to any of the outcomes of interest. While others have reported to the contrary, the strength of the associations have been weak. Being older and female were significantly, and independently, associated with a range of adverse outcomes, a finding supported by other research examining outcomes among people with varying injury types [22,24,25,32]. There is some evidence to suggest that poor outcomes for women are due to poorer care [33].

We examined a total of 12 pre-injury health and disability characteristics all of which appeared to be related to at least one of the outcomes (Table 3). When adjustment was made for other characteristics, there was no association of some factors with the outcome measures. Notable in this respect was a pre-existing disability. In contrast to the bivariate analyses (Table 3), which suggested a strong relationship with all six outcomes, it was only related to problems with mobility and pain or discomfort in the multivariate analyses (Table 5).

In contrast to this, physical activity, which was not related to any outcome in the bivariate analyses (Table 3), was related in the multivariate models to two outcomes (anxiety or depression and cognition) (Table 5). Contrary to what was hypothesized, physical inactivity prior to injury was protective in relation to these outcomes. It may be that people who were physically active before their injury were more acutely aware of their functional deficits after injury than those who did not exercise regularly.

This study has a number of strengths. As discussed above, it was not confined to those admitted to hospital or a trauma unit. Another strength is the wide range of potential risk factors studied. Consequently, we have reported a number of previously unreported and important associations (e.g. access to healthcare services). The results also demonstrate the value of including a wide range of injuries and examining very specific outcomes. Another strength is that we did not need to use proxy informants to obtain exposure and outcome information.

Participants in our study self-reported the nature of their injury. Even though participant descriptions may be informed by subsequent X-rays and tests, in many cases they are unlikely to be entirely error-free due to most participants' lack of anatomical knowledge. This is unlikely to be a problem for the injury groupings derived for our analyses. For example, whether a fracture was to the radius or ulna had no impact on which injury region (i.e. upper extremity) it was coded to. Irrespective of these points, what the patient believes is the nature of their injury and their understanding of consequences may have important implications for the manner in which they deal with the rehabilitation process.

We asked study participants, on average, three months after their injury to recall their EQ-5D status both prior to injury, and again at the time of the interview. We adjusted for both the pre-injury EQ-5D score and time between injury and interview in our analyses. This approach raises the potential for recall bias, with peoples' pre-injury self-assessment being influenced by their present status. There is surprisingly little published empirical research reporting these potential effects, and none, to our knowledge, specifically on injured people using the EQ-5D as an outcome measure. The most relevant study to the present investigation is that of 104 Italian patients with planned admissions to an intensive care unit (ICU). Their EQ-5D status was assessed on ICU admission. Three and six months later people were asked again to recall their admission status. The results showed that correlation between EQ-5D admission scores and follow-up recalled scores was very good [34]. Using data for 1015 individuals in the York Health Survey, a cohort of individuals identified from the patient list of a York general practice, Macran has shown that most individuals are able to accurately recall their health status in terms of EQ-5D eighteen months later [35]. While these two studies had samples which are not directly comparable to that used in our study, the results nevertheless offer some reassurance that if there is bias, it is likely to be small.

Injury victims seeking compensation for injury outcomes have, in theory, an incentive to inflate the quality of their health status prior to injury thus exaggerating the negative impact of the injury on their health status, which in turn could make them eligible for a higher level or duration of rehabilitation or compensation benefits. A strength of the present investigation was that all measurement was conducted completely independently of the ACC and any clinicians associated with the care of the injured person. Information provided to participants stressed the fact that the study was independent of all service providers and that under no circumstances would any information they provided be shared with anyone outside the research group.

## Conclusion

Our findings show that few socio-demographic factors are associated with adverse outcomes. Most notably, females had adverse scores on all six outcomes. Of the health and disability characteristics, having two or more prior chronic illnesses was associated with adverse outcomes; whereas being less physically active before injury appears to be protective. Among injury and health care factors, admission to hospital, self-perceived threat of disability and trouble accessing health services were consistently associated with the six outcomes studied.

In 2007, guidelines for the conduct of follow-up studies measuring injury-related disability were published [1]. These guidelines represented the views of the European Consumer Safety Association working group on injury-related disability. The guidelines imply that injury outcome studies should be confined to those with major trauma and that this should be defined in terms of a severity threshold based on a threat to life measure (e.g. ISS > 15). Whilst outcomes for major trauma cases are more likely to be adverse and sustained, our investigation has demonstrated that many injuries, which by service utilization criteria alone (e.g. not admitted to hospital) can be assumed not to represent a significant threat to life, do nevertheless represent significant threat of disability, albeit relatively short-term after injury.

## Competing interests

The authors declare that they have no competing interests.

## Authors' contributions

JL, SD, and GD jointly planned the scope and content of the paper. GD undertook the statistical analyses. JL, SD, GD & EW were actively involved interpreting the results, contributing to various drafts and approving the final manuscript.
